# Logwood as an Antiseptic

**Published:** 1862-10

**Authors:** 


Logwood as an Antiseptic.—Dr. W. N. Coté, the intelligent Paris correspondent of the *British American Journal*, says, in a recent communication:

"Your readers may recollect the interest excited among professional men when Dr. Demeaux discovered the antiseptic qualities of coal tar, a mixture of which with plaster being applied to the most fetid sores, will at once dispel the offensive smell, and at the same time contribute to the speedy cure of the part affected. The Academy of Sciences has now received a paper from Dr. Desmartis, announcing that logwood or campeachy (*Hœmatoxylon Campeachianum*) possesses the same valuable property, and in a much higher degree. This fact was discovered by accident; Dr. Desmartis had several cancerous patients under his care, all presenting large ulcerous sores, emitting a most nauseous smell. An astringent being considered expedient, a pomatum composed of equal parts of logwood and hog's lard was applied to these sores; whereupon, to the doctor's surprise, the fetor disappeared completely, and the emission of pus was considerably attenuated. To complete the evidence, he suspended the use of the pomatum for a few hours only, when the offensive emanations immediately recommenced, and the purulent secretion became again abundant. Logwood, as he has now ascertained, causes gangrene, especially that of hospitals, to disappear, as if by enchantment. Dr. Desmartis has also found it efficacious in preventing or stopping the erysipelas which often occurs after amputation, or the infliction of other wounds, and is a source of constant anxiety to the surgeon. It entirely removes the putridity of ulcerous cancers emitting characteristic effluvia, and, in short, of the most fetid sores. This substance also possesses the advantage of being capable of mixture with hæmostatic remedies, such as ergotine, perchloride of iron, persulphate of iron, etc.; it may also be used as a powder and a lotion. The extract of hæmatoxylon, which is much used in dyeing, and is very cheap, is soluble only in warm water."—*Med. and Surg. Reporter.*

